# Physical Properties of Concrete Containing Graphene Oxide Nanosheets

**DOI:** 10.3390/ma12101707

**Published:** 2019-05-26

**Authors:** Yu-You Wu, Longxin Que, Zhaoyang Cui, Paul Lambert

**Affiliations:** 1 School of Transportation, Civil Engineering and Architecture, Foshan University, Foshan 528000, China; 2 Department of Civil Engineering, Chengdu University of Technology, Chengdu 61000, China; qlx142316@163.com (L.Q.); cuizhayang0421@foxmail.com (Z.C.); 3 Materials and Engineering Research Institute, Sheffield Hallam University, Sheffield S1 1WB, UK; p.lambert@shu.ac.uk

**Keywords:** ordinary Portland cement, concrete, physical properties, graphene oxide

## Abstract

Concrete made from ordinary Portland cement is one of the most widely used construction materials due to its excellent compressive strength. However, concrete lacks ductility resulting in low tensile strength and flexural strength, and poor resistance to crack formation. Studies have demonstrated that the addition of graphene oxide (GO) nanosheet can effectively enhance the compressive and flexural properties of ordinary Portland cement paste, confirming GO nanosheet as an excellent candidate for using as nano-reinforcement in cement-based composites. To date, the majority of studies have focused on cement pastes and mortars. Only limited investigations into concretes incorporating GO nanosheets have been reported. This paper presents an experimental investigation on the slump and physical properties of concrete reinforced with GO nanosheets at additions from 0.00% to 0.08% by weight of cement and a water–cement ratio of 0.5. The study demonstrates that the addition of GO nanosheets improves the compressive strength, flexural strength, and split tensile strength of concrete, whereas the slump of concrete decreases with increasing GO nanosheet content. The results also demonstrate that 0.03% by weight of cement is the optimum value of GO nanosheet dosage for improving the split tensile strength of concrete.

## 1. Introduction

Concrete made from ordinary Portland cement is one of the most widely used construction materials [1]. As a structural material, concrete is desirable because of its excellent compressive strength. However, concrete has poor ductility, with low tensile strength and flexural strength, and poor resistance to crack formation [1]. Many attempts have been made to enhance its properties and performance by adding supplementary cementitious materials (e.g., fly ash, blast-furnace slag, etc.) and fibers (e.g., glass and steel), but they fail to adequately enhance its physical properties and durability at the nanoscale [2,3]. 

Ordinary Portland cement is the principal binder holding sand and coarse gravels or crushed rocks together to produce concrete when water is added to initiate the hydration. It is reported that the global production of cement has exceeded 3600 million tonnes annually with more than 55% from China since 2011 [4]. The cement industry accounted for 15% to 20% of China’s PM2.5 emissions, 3% to 4% of sulfur dioxide emissions, and 8% to 10% of nitrogen oxides emissions [5], resulting in a drive to cut air pollution deriving from the cement industry in China. 

Advancements in nanotechnology can generate opportunities to improve the properties and performance of cement by incorporating nanomaterials [6]. Studies have demonstrated that the addition of 0.05% graphene oxide (GO) nanosheets (by weight of cement) can effectively increase the compressive strength and flexural strength of ordinary Portland cement paste by 15% to 33% and 41% to 58%, respectively, and it is suggested that this may be associated with the enhanced mechanical interlocking, interaction between the microcracks and the GO nanosheets, promotion of the hydration process, and the formation of powerful interfacial forces between carboxylic groups and hydration products [7]. Such observations strongly indicate the role of the GO nanosheet can potentially play in the nano-reinforcement in cement-based composites. To date, the majority of studies have focused on cement paste and cement mortar, including their physical properties [8,9], durability [10,11,12,13], and rheological behavior [14,15]. Limited results have been reported on the physical properties of ultra-high strength concrete (UHSC) incorporating with GO nanosheets [16] and ultra-high-performance concrete (UHPC) containing graphene nanoplatelets (GNPs) [17] and graphene [18]. Lu and Ouyang [16] demonstrated that the fluidity of UHSC decreased, and its flexural and compressive strengths improved with the addition of GO nanosheets from 0.00% to 0.03% by weight of cement. The compressive strength of UHSC incorporating 0.01% at 28 days enhanced by 7.82% compared with that of UHSC without GO nanosheets. Meng and Khayat [17] verified that when the GNPs content is less than 0.05%, the high-range water reducer (HRWR) demand decreased, meaning the use of GNPs improved the flowability decrease, whereas an adverse effect on flowability was obtained with GNPs content greater than 0.05%. And the authors also demonstrated the use of GNPs did not have a significant effect on the compressive strength of UHPC with an increase of 5.2% to 5.7% while the use of 0.3% GNPs increased the flexural strength and the tensile strength by 39% to 59% and 40% to 45%, respectively, dependent on the dimension and specific surface area of GNPs. Dimov and Craciun et al. [18] concluded that the compressive strength and the flexural strength of UHPC can increase up to 146% and 79.5%, separately. However, it should be noted that relatively little work has been concerned with ordinary concrete incorporating GO nanosheets. It is, therefore, apparent that further research in this area is required [3,19,20]. 

In this study, the effects of GO nanosheets on the slump and physical properties of concrete are experimentally investigated. The GO nanosheet content is at additions of 0.00%, 0.02%, 0.03%, 0.04%, 0.06%, and 0.08% by weight of cement under a water–cement ratio of 0.5. The evaluated physical properties of concrete include the compressive strength, flexural strength, and split tensile strength. Additionally, the optimum value of GO nanosheet dosage for improving the split tensile strength of concrete is also discussed.

## 2. Materials and Methods 

### 2.1. Materials

**Figure 1:**
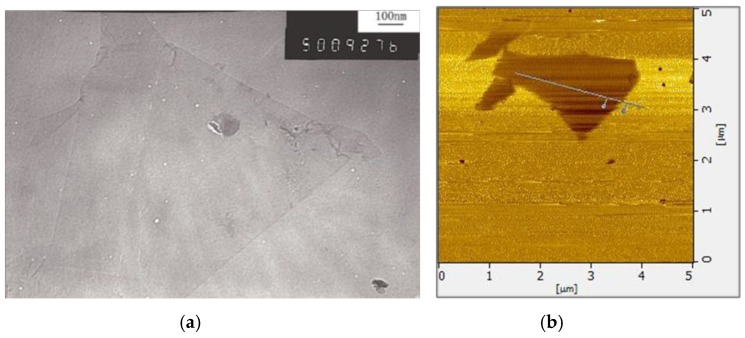
Images of typical GO nanosheet. (**a**) transmission electron microscopy (TEM), (**b**) atomic force microscope (AFM).

Ordinary Portland cement (OPC) type 42.5R was used for all the concrete mixes, and its chemical composition is shown in Table 1. Fine aggregate (FA) and coarse aggregate (CA) were natural river sand with a fineness modulus of 2.74 and crushed quartz with a size range from 5 mm to 20 mm, respectively. A polycarboxylate-based superplasticizer (PCs) was used to improve the workability of concrete and was provided by Sichuan Zhaohui Xincheng New Materials and Technology Ltd., Chengdu, China. The GO nanosheet was used as a water dispersion solution which was synthesized by using a modified Hummers method [21] at Chengdu Institute of Organic Chemistry, China Academy of Sciences, Chengdu, China. The main parameters of the GO nanosheet are shown in Table 2. Figure 1a shows a transmission electron microscopy (TEM) image of a typical GO nanosheet with wrinkled and folded features. Figure 1b shows an atomic_force microscope (AFM) image of a typical GO nanosheet with irregular shapes. 

### 2.2. Concrete Mix and Specimen Preparation

Six concrete mixes were prepared. The concrete mix proportions are shown in Table 3 and are in accordance with the relevant specification [22] and code [23]. All mixes had a water to cement ratio (w/c) of 0.5. The GO nanosheets were added to the CGO0, CGO2, CGO3, CGO4, CGO6, and CGO8 at additions of 0.00%, 0.02%, 0.03%, 0.04%, 0.06%, and 0.08% by weight of cement. The total water content was 168 kg/m^3^, which included the mixing water, GO nanosheet water dispersion solution, and PCs solution.

Both the fine aggregate (FA) and coarse aggregate (CA) were air dried in the laboratory environment. Cement was first mixed with the fine aggregate in a twin shaft concrete mixer for two minutes, and then the coarse aggregate was added before mixing for a further two and a half minutes at moderate speed. The potable mix water was pre-mixed in a separate vessel with the PCs solution and mechanically stirred for one and half minutes using a hand-mixer, which was followed by adding the GO nanosheet water dispersion solution and further stirring for another one and half minutes at a moderate speed. The pre-mixed solution was added to the dry mixed cement and aggregates and then mixed for two and a half minutes at moderate speed. The freshly mixed concrete was poured into pre-oiled molds and compacted on an electric concrete vibration table after which the specimens were covered with a polyethylene sheet and cured in the laboratory for twenty-four hours. Finally, the specimens were demolded and cured at a relative humidity greater than 95% and a temperature of 21 ± 2 °C until tested.

### 2.3. Physical Property Tests and Slump Test

**Figure 2:**
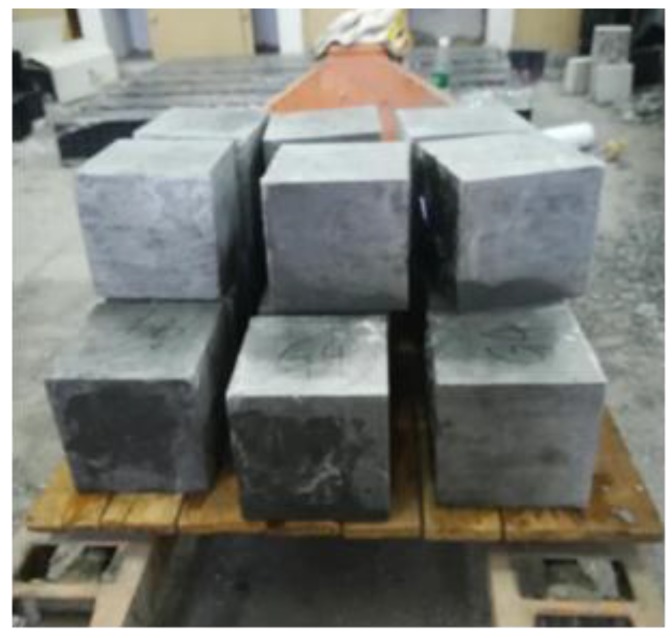
Concrete specimens for the compressive strength test.

The compressive strength, flexural strength, and split tensile strength of specimens were measured on day 7, day 14, and day 28. The concrete specimens were tested in accordance with the GB/T50081-2002, Standard for Test Method of Mechanical Properties on Ordinary Concrete [24]. The specimens for each test are listed in Table 4. A selection of specimens for compressive strength test are shown in Figure 2. The loading rate employed was 0.6 MPa/s. It should be noted that due to the smaller, non-standard size of the specimens used, the results from the compressive strength, flexural strength, and split tensile strength tests were reduced by multiplying the conversion coefficients of 0.95, 0.85, and 0.85, respectively, according to the GB/T50081-2002 Standard [24]. The slump test was carried out in accordance with the GB/T50080-2002 Standard [25], which was employed to assess the workability of concrete.

## 3. Results and Discussion

### 3.1. Workability

**Figure 3:**
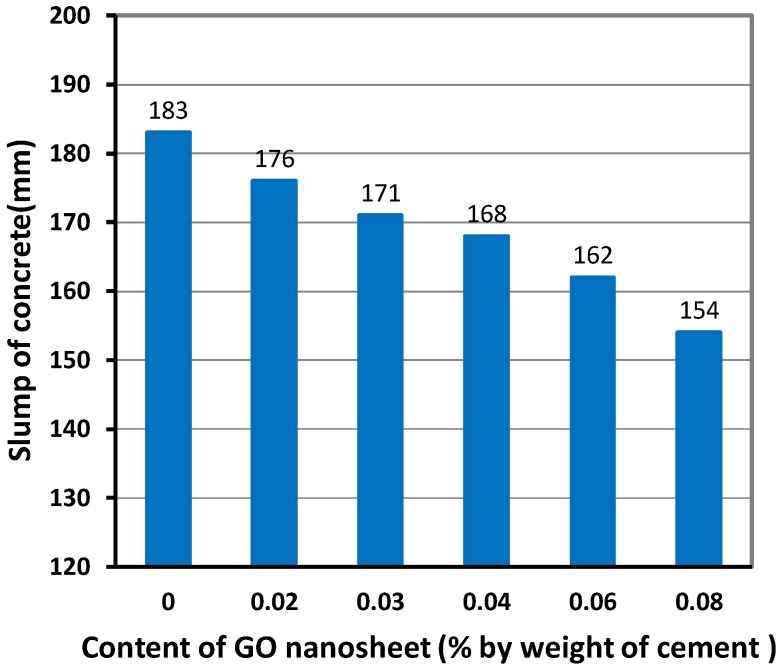
Slump of concrete.

The results obtained from the slump test are shown in Figure 3. It can be seen that the values of slump obtained for concrete specimens with GO nanosheets (GCO02, GCO3, GCO4, GCO6, and GCO8) are lower than that of concrete without GO nanosheets (GCO0). It is also observed that the value of the slump of concrete decreased with the increase of the dosage of GO nanosheets. A similar result has also been reported in a previous study where an ultra-high strength concrete (UHSC) incorporating GO nanosheets was investigated [16]. This may be caused by the large surface area of the GO nanosheet, resulting in a decrease in the availability of water in the fresh mix from wetting [7,16]. It is possible to improve the slump of conventional mixes by using fly ash [15] and PCs [26]. However, Meng and Khayat [17] verified in their study that when the GNPs content is less than 0.05%, the high-range water reducer (HRWR) demand decreased, meaning the use of the GNPs improved the flowability of the UHPC, whereas an adverse effect on its flowability was obtained with GNPs content greater than 0.05%. This suggests that the workability of the UHPC is affected by both the additive and the GNPs. It is, therefore, necessary to perform further studies to better understand the influence of GO nanosheets on the workability of ordinary concrete.

### 3.2. Compressive Strength

**Figure 4:**
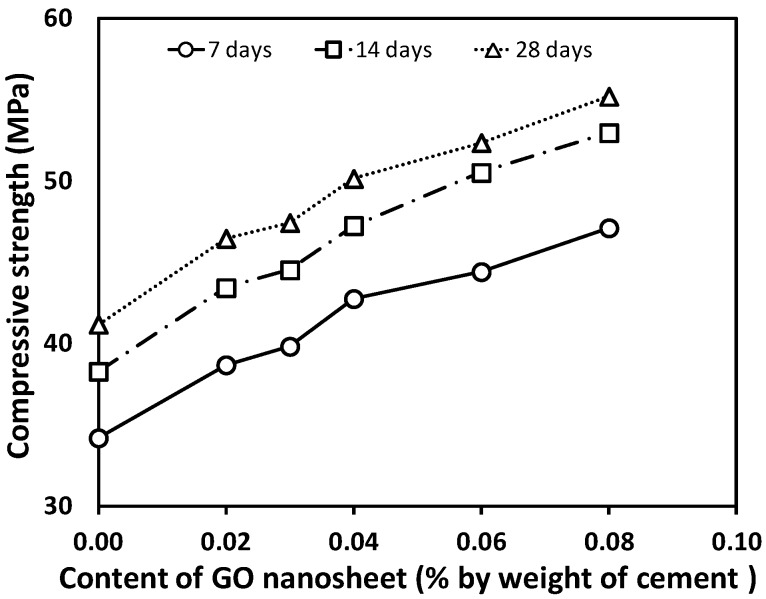
Compressive strength of concrete with varying graphene oxide (GO) content.

The results of the compressive strength tests for concrete specimens with ages of 7, 14, and 28 days under the different GO contents are shown graphically in Figure 4. Each value presented is the average of three test results. It is observed that the concrete specimens containing the GO nanosheets (GCO02, GCO3, GCO4, GCO6, GCO8) generally have higher compressive strength compared with the concrete with no addition (GCO0). When the content of GO nanosheets increased from 0.02% to 0.08%, the 28-day compressive strength increased from 46.47 MPa to 55.22 MPa, representing an increase from 12.84% to 34.08% when compared to the concrete specimens without GO nanosheets (GCO0). This confirms that the compressive strength of the concrete is enhanced by an increase in the content of GO nanosheets from 0.02% to 0.08% for the water–cement ratio of 0.5. Based on the study on the cement paste reinforced with GO nanosheets, this may be the result of enhanced mechanical interlocking, intense interaction between the microcracks and the GO nanosheets, promotion of the hydration process or the formation of powerful interfacial force between carboxylic groups and hydration products [7]. For the UHPC enhanced with GNPs, it can be attributed to the “bridging effect” of GNPs for microcracks and the “filler effect” for accelerating the hydration reactions of the cementitious materials [17,27]. However, such a mechanism for the UHSC incorporating GO nanosheets was not fully examined by FE-SEM [16], resulting in further study being required.

**Figure 5:**
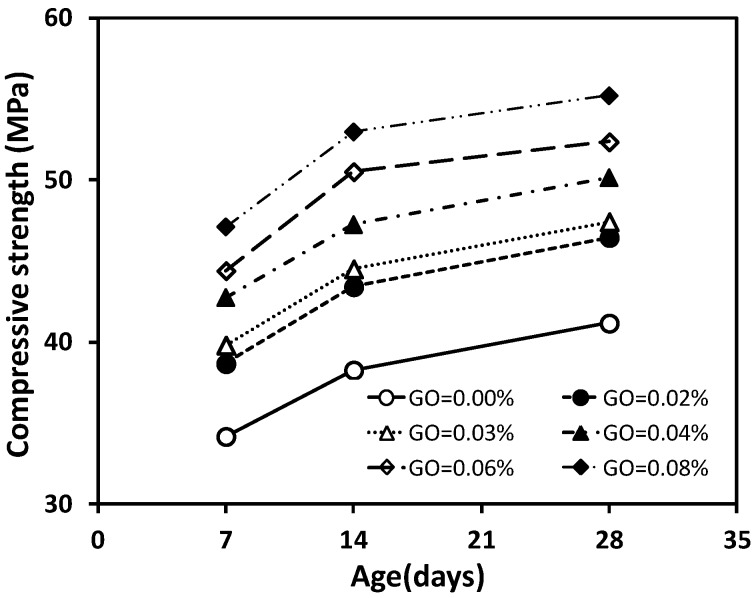
Compressive strength of concrete at different ages.

The trend in the compressive strength of the concrete specimens with age is shown in Figure 5. This trend is different from that observed for the UHSC with additions of GO nanosheets in an earlier study [16], where it was suggested that the values of compressive strength and flexural strength of concrete from an addition 0.01% were greater than those from an addition of 0.03%. One explanation may be a lower water-cement ratio of 0.2 [16]. However, the compressive strength of concrete is affected by many factors, such as the water-cement ratio, raw material types, and complementary materials and admixtures [1]. 

### 3.3. Flexural Strength and Relationship between Compressive and Flexural Strength 

**Figure 6:**
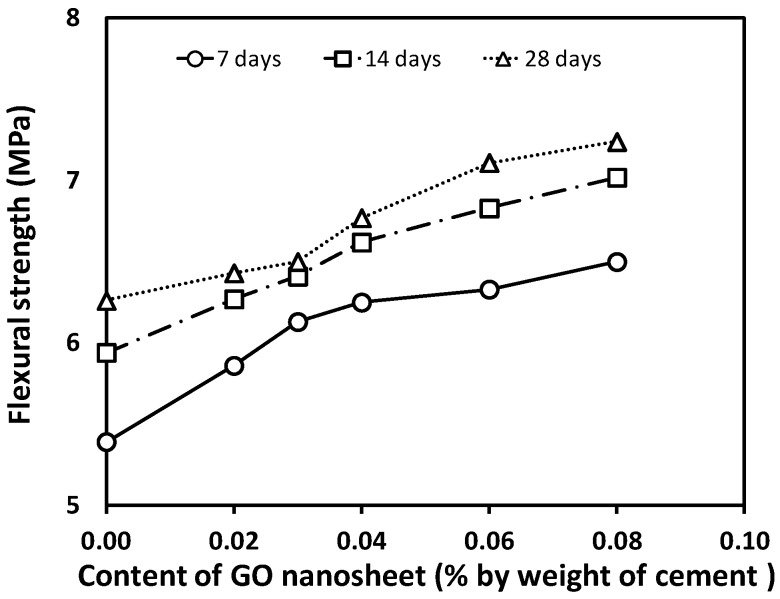
Flexural strength of concrete containing varying GO content.

**Figure 7:**
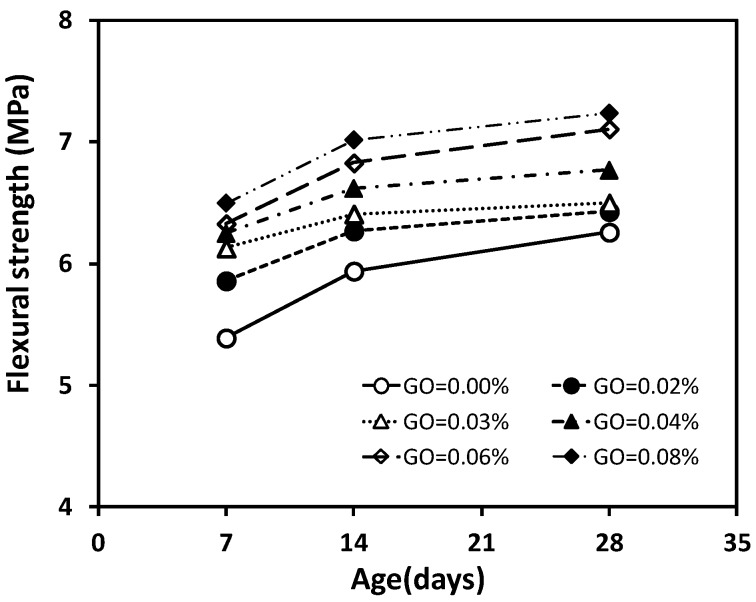
Flexural strength of concrete at different ages.

The flexural strength results of the concrete specimens at 7, 14, and 28 days under the different GO contents are shown graphically in Figure 6. Each value presented is the average of three test results. It is apparent that the addition of GO nanosheets improves the flexural strength of all concrete specimens with GO nanosheets (GCO2, GCO3, GCO4, GCO6, and GCO8). The flexural strength enhances in a range from 2.77% to 15.60% at 28 days when the content of GO nanosheets increases from 0.02% to 0.08%. However, the rate of increase in the flexural strength is generally less than that of the compressive strength for concrete with GO nanosheet additions from 0.02% to 0.08%. Additionally, it is also observed that the increasing rate before 14 days is greater compared with that from 14 to 28 days for a given GO nanosheets addition from 0.02% to 0.08%. The trend of the flexural strength of the concrete specimens versus age at various GO nanosheets additions is shown in Figure 7.

**Figure 8:**
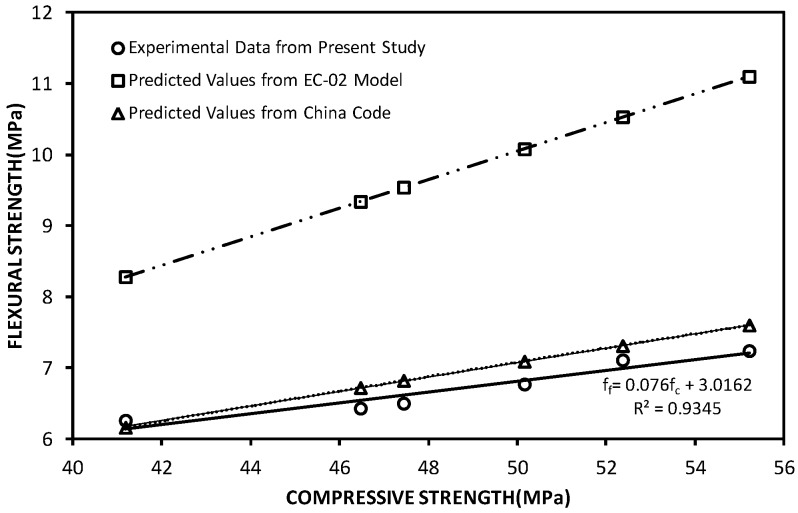
Relationship between compressive strength and flexural strength of concrete.

It is widely accepted that the compressive and flexural strengths of concrete are related. Various empirical relationships for predicting the flexural strength have been proposed, such as the European [28] and China [29] codes. Table 5 shows the values of flexural strength and compressive strength of the concrete specimens at 28 days, which were obtained from both the current experimental study and the predicted models. It is apparent that there are differences between the experimental and predicted values, most notably with those in EC-02, as illustrated in Figure 8 and Table 5. 

Empirical models for predicting the flexural strength of concrete containing GO nanosheets based on its compressive strength have not been reported in previous studies. Building on the experimental data of the flexural and compressive strengths listed in Table 5, and fitting the data (see Figure 8), an empirical function with coefficients of determination *r*^2^ = 0.9345 was developed:

(1) $$f_{f}=0.076f_{c}+3.0612$$

where *f_c_* is the compressive strength at 28 days and *f_f_* is the flexural strength at 28 days. It should be noted that this relationship may be subject to update when more data become available.

### 3.4. Split Tensile Strength 

**Figure 9:**
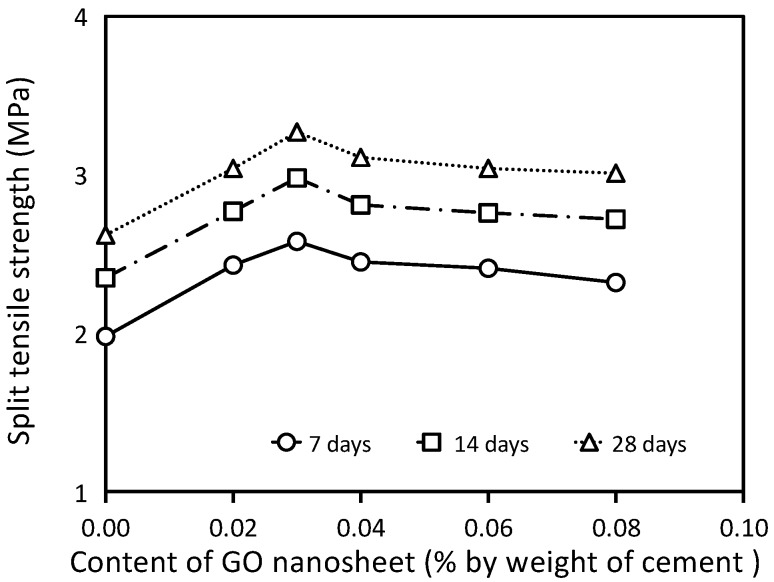
Split tensile strength of concrete at different ages.

The split tensile strength results for the concrete specimens at 7, 14, and 28 days are shown in Table 6 and Figure 9. Each value presented is the average of three test results. The results show that the split tensile strength of the specimens with a water-cement ratio of 0.5 generally increases with increasing GO nanosheets content from 0.02% to 0.08%. It can also be seen that the split tensile strength of the concrete increases when the GO nanosheet dosage increases from 0.0% to 0.03%, which is followed by a gradual decrease of such strength with the increasing GO nanosheet dosage from 0.04% to 0.08%. Furthermore, it is observed that the split tensile strength of concretes with GO nanosheet content of 0.02% and 0.06% are comparable. It is important to note that the concrete specimen with a GO nanosheet dosage of 0.03% (GCO3) shows the greatest enhancement, indicating that 0.03% is the optimum value of GO nanosheet dosage for improving the split tensile strength of concrete with a water-cement ratio of 0.5.

## 4. Conclusions

The present study has led to the following conclusions:

(1) The slump of concrete containing GO nanosheets decreases with the addition of GO nanosheets from 0.02% to 0.80% by weight of cement under a water to cement ratio of 0.5. However, the workability of concrete is affected by both the additive and the GNPs. Therefore, further study is needed to better understand the influence of GO nanosheets on the workability of concrete.
(2) The compressive strength of the concrete is enhanced by an increase at the level of GO nanosheets from 0.02% to 0.08% for the water–cement ratio of 0.5. It may be associated with many factors, such as the promotion of the hydration process, the “bridging effect” of GO nanosheets for microcracks, etc. This will be subjected to further examination by SEM.
(3) The addition of GO nanosheets improves the flexural strength of concrete in a range from 2.77% to 15.60% at 28 days when the content of GO nanosheets increases from 0.02% to 0.08%. However, the rate of increase in the flexural strength is generally less than that of the compressive strength.
(4) The split tensile strength of the specimens with a water–cement ratio of 0.5 generally increases with increasing GO nanosheets content. The results also indicate that 0.03% is the optimum value of GO nanosheet dosage for improving the split tensile strength of the concrete specimens with a water–cement ratio of 0.5.

## Figures and Tables

**Table 1:** Chemical composition of cement (% by weight).

Component	CaO	SiO_2_	Al_2_O_3_	Fe_2_O_3_	MgO	K_2_O	Na_2_O	SO_3_
Content (%)	65.16	21.25	4.21	3.35	2.90	0.97	0.50	0.72

**Table 2:** The composition and dimensions of graphene oxide nanosheet.

Items	Carbon (%)	Oxygen (%)	The Length/Width (μm)	Thickness (nm)
Value range	45–60	40–55	2–10	1–1.5

**Table 3:** Concrete mixture proportions.

Mix ID	Total Water (kg/m^3^)	Cement (kg/m^3^)	FA (kg/m^3^)	CA (kg/m^3^)	PCs (kg/m^3^)	GO (%)	GO (kg/m^3^)
GCO0	168	336	626	1270	6.72	0.00	0.0000
GCO2	168	336	626	1270	6.72	0.02	0.0672
GCO3	168	336	626	1270	6.72	0.03	0.1008
GCO4	168	336	626	1270	6.72	0.04	0.1344
GCO6	168	336	626	1270	6.72	0.06	0.2016
GCO8	168	336	626	1270	6.72	0.08	0.2688

**Table 4:** Size of specimens.

Test	Size Used (mm)	Standard Size (mm)	Conversion Coefficients
Compressive strength	100 × 100 × 100	150 × 150 × 150	0.95
Flexural strength	100 × 100 × 400	150 × 150 × 550	0.85
Split tensile strength	100 × 100 × 100	150 × 150 × 150	0.85

**Table 5:** Flexural strength of concrete from the current study and as predicted by models (N/mm^2^)^1^.

Mix ID	Compressive Strength on Day 28 (*f_c_*)	Flexural Strength (*f_f_*)		
		Present Study	EC-02 [28] *f_f_* = 0.201*f_c_*	China Code [29] *f_f_* = 0.435 *f_c_* ^0.713^
GCO0	41.18	6.26	8.28	6.16
GCO2	46.47	6.43	9.34	6.72
GCO3	47.44	6.50	9.54	6.82
GCO4	50.16	6.77	10.08	7.09
GCO6	52.37	7.11	10.53	7.31
GCO8	55.22	7.24	11.10	7.60

^1^*f_c_* = compressive strength at 28 days. *f_f_* = flexural strength at 28 days.

**Table 6:** Split tensile strength of concrete with varying GO nanosheet contents.

Mix ID	GO (%)	Split Tensile Strength (MPa)			Increase at 28 Days (%)
		7 Days	14 Days	28 Days	
GCO0	0.00	1.98	2.35	2.62	0.00
GCO2	0.02	2.43	2.77	3.04	16.18
GCO3	0.03	2.58	2.98	3.27	24.81
GCO4	0.04	2.45	2.81	3.11	18.75
4CO6	0.06	2.41	2.76	3.04	15.95
GCO8	0.08	2.32	2.72	3.01	14.79
